# Detection of Flying Metal Bodies Based on Photoelectric Composite Sensing

**DOI:** 10.3390/s23062926

**Published:** 2023-03-08

**Authors:** Weitao Gao, Tiehua Ma, Changxin Chen, Chenbin Wang, Na Feng

**Affiliations:** 1 State Key Laboratory of Dynamic Testing Technology, North University of China, Taiyuan 030051, China; 2 School of Electrical and Control Engineering, North University of China, Taiyuan 030051, China

**Keywords:** photoelectric, eddy current, composite sensing, flying metal body, detection

## Abstract

In order to reduce the impact of the environment on the accuracy and sensitivity of detection, and to meet the requirements of concealment from detection and being lightweight, a technology for detecting flying metal objects based on photoelectric composite sensors is proposed. The method first analyzes the target’s characteristics and detection environment, and then compares and analyzes the methods for detecting typical flying metal objects. On the basis of the traditional eddy current model, the photoelectric composite detection model that meets the requirements of detecting flying metal objects was studied and designed. For the problems of the short detection distance and the long response time of the traditional eddy current model, the performance of the eddy current sensor was improved to meet the requirements of detection through optimizing the detection circuit and coil parameter model. Meanwhile, to meet the goal of being lightweight, an infrared detection array model applicable to flying metal bodies was designed, and simulation experiments of composite detection based on the model were conducted. The results show that the flying metal body detection model based on photoelectric composite sensors met the requirements of distance and response time for detecting flying metal bodies and may provide an avenue for exploring the composite detection of flying metal bodies.

## 1. Introduction

The technology of metal detection is closely related to the development of electronic technology. In the late 1960s and early 1970s, with the rapid development of semiconductor electronics, very lightweight metal detectors appeared, such as the body scanning device used by public security departments. Around the 1990s, with the development of large-scale integrated circuits and microprocessors, metal detectors entered an upgrading period and began to realize automation and intelligence, with data processing functions, including storage, calculation, analysis and comparison, display, and automatic alarms. At present, the application of metal detection technology is very wide and deep. Militarily, it can be used for mine clearance; for archaeology, we can use it find the tombs where metal objects are buried, such as treasures of gold and silver in the tombs. In mineral exploration, it can be used to detect and discover natural gold particles and gold nuggets. Metal detectors are also widely used for target detection in airports, high-speed railway stations, subway stations, and other important places [1].

**Figure 1:**
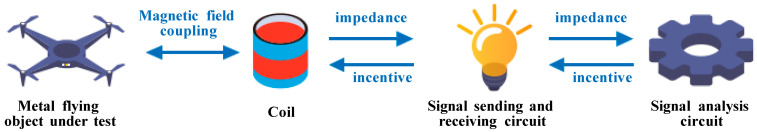
Schematic diagram of eddy current detection of metal flying body.

The detection of flying metal objects has always been an important subject in the fields of aerospace, missile defense, and military operations. At present, the detection of moving targets such as metal warheads, unmanned aerial vehicles (UAV), and vehicles is affected by the speed, attitude, infrared characteristics, and distance of the measured target, and most of the composite detection technology based on multi-sensor fusion is used to make up for the lack of accuracy and robustness of a single detection unit. In the composite detection process of warheads, UAV, and other flight targets, the commonly used detection elements such as lasers and infrared and millimeter wave radar have strong radiation characteristics and high-power consumption, and are restricted when applied to mobile platforms, whereas a large-radius eddy current sensor has better concealment ability and weaker radiation characteristics. In the process of detecting flying metal objects from a mobile platform, the combined detection method of an infrared array and eddy currents can greatly reduce the system’s cost, power consumption, and radiation characteristics [2]. With the development of metal detection technology based on electromagnetic fields, early examples used the electromagnetic signals constantly around metal to detect moving targets [3]. In this study, a technology used for detecting flying metal bodies based on photoelectric composite sensing was proposed. This method makes comprehensive use of photoelectric and eddy currents to form a two-stage detection network. Through use of the detection mode of composite sensing, the incoming target can be detected actively and responded to quickly. Before the incoming target hits tanks and armored vehicles, a jet can be launched to destroy and intercept the incoming target, realizing active protection. Here, active protection refers not only to the active protection system of armored vehicles but also to any system that needs protection. The active detection technology proposed in this study can be used as the final protective measure. When the technology is mature, it can be applied to the modular active armor on armored vehicles to reduce damage on the battlefield and improve the chances of victory. Moreover, compared with infrared, laser, millimeter wave radar, and other detection methods commonly used in the modern battlefield, this detection method using eddy currents and photoelectric composite sensing has better concealment ability and weaker infrared characteristics. This method of detection mainly relies on the joint judgment of the eddy current detector and photodetector, which can minimize misjudgment and missed judgment, so it has far-reaching significance. A schematic diagram of the process of detecting flying metal bodies by using eddy currents is shown in Figure 1.

## 2. Literature Review

With the development of technologies for detecting flying metal objects based on electromagnetic fields, earlier work has studied the technology of detecting moving metal targets using electromagnetic signals [4]. Yin et al. proposed an improved algorithm for analyzing voltage or impedance across long ideal notches of tilting driver-pickup eddy current sensors [5]. Eddy current sensors have high measurement accuracy and strong robustness, and their output signal is not affected by environmental factors when detecting the characteristics of an object’s motion. Structural designs and model simulations of application scenarios of eddy current sensors have been carried out [6,7]. He Ping et al. studied the effect of distance on space-based infrared detectors on adjacent-space targets. A range model based on the representation contrast was constructed, and the process of solving the stepwise approximation was given. On the basis of actual aircraft and backgrounds, the range of the space-based infrared detector was simulated [8]. Liping Lu et al. analyzed the measurement principle of the infrared photoelectric detection system; established a model for calculating the radiation energy of the photodetector’s surface background, optical system, and target; further analyzed the influence of the wavelength of the photodetector, the slit aperture, and the effective radiation area of the target on the radiation energy; and obtained a model for calculating the contrast of the photodetector’s surface target and the background. At the same time, a model of the detection range of the system was derived and combined with the modulation transfer function of the system to establish an improved model for calculating the detection range in an environment with a complex background [9].

## 3. Research Method

### 3.1. Design of the Solution Process

**Figure 2:**
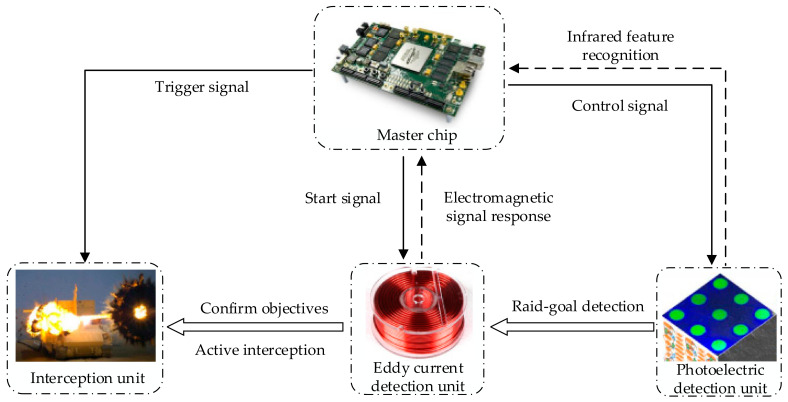
Detection system workflow diagram.

The workflow diagram of the detection system is shown in Figure 2, and the main steps are as follows:

(1) When the incoming target enters the detection range of the detection device, the received infrared characteristics of the infrared detection array is used to make a comprehensive judgment of the location of the incoming target.
(2) When the incoming target is close to the coil, eddy currents are generated on the incoming metal target, and the reverse magnetic field generated by the incoming target eddy currents causes a change in the magnetic flux of the coil.
(3) The magnetic flux of the surface of the coil can be confirmed by detecting the degree of change in the magnetic flux of the surface of the coil and the distance of the incoming target from the detection device.
(4) When the incoming target reaches a preset value of distance, the interception device is released to intercept and destroy the incoming target.

### 3.2. Analysis of the Detection Environment and Design Scheme

#### 3.2.1. Analyses of the Characteristics of the Target and the Detection Environment

All substances emit electromagnetic radiation to their surroundings when they have a certain temperature (that is, when the temperature is above the thermodynamic temperature of 0 K or −273 °C). At room temperature, infrared radiation is the main source of the spontaneous radiation of objects [10]. Infrared radiation, also known as infrared, is affected not only by temperature but also by many other factors. The basic law of the infrared radiation of an ideal blackbody can be defined as Plank’s blackbody radiation law, Wein’s displacement law and the Stefan–Boltzmann law [11].

(1) Plank’s blackbody radiation law

Plank’s blackbody radiation law reveals the distribution of a blackbody’s radiation energy as a function of wavelength and temperature, and describes the variation in the radiation flux density $W_{B}(\lambda ,T)$ with the wavelength *λ* and the thermodynamic temperature *T* at different temperatures. Its mathematical expression is

(1) $$W_{B}(\lambda ,T)=\frac{c_{1}}{\lambda^{5}}\frac{1}{e^{c_{2/\lambda T}}-1}$$

where $W_{B}(\lambda ,T)$ is the blackbody’s spectral radiation flux density, *λ* is the wavelength (m), *T* is the thermodynamic temperature of the blackbody, $c_{1}=3.742\times 10^{-16}\;W\cdot m^{-2}$ is the first radiation constant, and $c_{2}=1.4388\times 10^{-2}\;m\cdot K$ is the second radiation constant.

(2) $$I_{B}(\lambda ,T)=\frac{C_{1}}{\pi \lambda^{5}}\frac{1}{e^{c_{2}/\lambda T}-1}=\frac{W_{B}(\lambda ,T)}{\pi }$$

where $I_{B}\left(\lambda ,T\right)$ is the blackbody’s spectral radiance.

(2) Wein’s displacement law

The energy of blackbody radiation varies with the wavelength. Wein’s displacement law gives the relationship between the peak value of spectral radiation *λ_M_* of an ideal blackbody and the thermodynamic temperature *T*. The mathematical expression is as follows:

(3) $$\lambda_{M}T=2897.8\pm 0.4\;(\mu m)$$

where *λ_M_* is the peak value of spectral radiation of an ideal blackbody, *T* is the thermodynamic temperature.

(3) Stefan–Boltzmann law

Flux density is a physical quantity that represents the blackbody’s ability to radiate. The Stefan–Boltzmann law gives the relationship between the flux density w of an ideal blackbody and the thermodynamic temperature *T*, and its mathematical expression is as follows

(4) $$W=\sigma T^{4}$$

where $\sigma =5.67\times 10^{-8}\;W\cdot m^{-2}\cdot K^{-4}$ is the Stefan–Boltzmann constant (the blackbody radiation constant) and *W* is the power emitted per unit of blackbody area (radiant flux density).

Typical flying metal bodies, such as artillery shells and rockets, have the characteristics of high temperature and high thermal radiation intensity in the process of high-speed movement, and have the infrared radiation characteristics of small infrared military targets [12,13].

#### 3.2.2. Analysis of the Characteristics of the Detection Environment

The imaging background of typical military infrared targets (artillery shells, rockets) is mainly the natural background, such as the sky and sea [14].

(1) Infrared radiation characteristics of the background sky

The ideal sky radiation consists of atmospheric radiation and sky radiation scattered by sunlight. Because of the differences in the atmospheric radiation and the scattering brightness corresponding to different elevation angles, the infrared wavelength of the sky radiation is less in the vicinity of 3–5 μm (middle wave infrared) and more in the vicinity of 8–12 μm (far infrared).

(2) Infrared radiation characteristics of the sea surface in the background

The ideal sea surface temperature is between 0 and 30 °C, and the peak wavelength of its radiation is between 8 and 12 μm (far infrared). The detection sensitivity, detection range, sensitivity, and effective response distance of photoelectric conversion devices will be greatly affected by changes in the brightness of the sky background when detecting flying metal objects with a natural background such as the sky and/or sea. Thus, the stability of the photoelectric detection system is affected. Therefore, it is not appropriate to use photoelectric conversion devices for the effective and reliable detection of flying metal objects in natural light such as sky and sea. The above analysis shows that the peak infrared wavelength radiated by the infrared background of the detection environment and the detection target is in two different infrared wavelengths, so the corresponding band can be used for the detection of high-speed flying metal bodies. The infrared wavelengths radiated by the environment are in the far infrared. The infrared wavelength of typical flying metal body radiation during the process of high-speed flight is medium wave, so in the detection of such objects, one can use a detector of the infrared wave (not actively emitting a light source or passively receiving infrared radiation) with the corresponding processing circuit to detect flying metal bodies.

### 3.3. Methods of Detecting Typical Flying Metal Bodies and a Comparative Analysis

The motion parameters of projectiles, rockets, and other typical flying metal bodies can be measured. These can be roughly divided into measurements of the displacement, the velocity, and the acceleration [15].

Displacement is a vector. When measuring displacement, the direction and magnitude of displacement should be confirmed. According to the principle of measurement, displacement measurement methods can be divided into: (1) the mechanical displacement measurement method, such as a tank level meter with a buoy to measure the change in a liquid’s level; (2) the electrical displacement measurement method, where the change in the displacement signal received by the displacement sensor is changed into an electrical signal, which is then transmitted through the corresponding conditioning circuit process to obtain the displacement; (3) the photoelectric displacement measurement method, in which the changes in the displacement value are transmitted by the photoelectric displacement sensor into changes in the electrical signals after the corresponding conditioning circuit processing, thus obtaining the displacement. For measuring the displacement of flying metal bodies, the commonly used methods are inductive displacement measurements, eddy current displacement measurements and photoelectric position sensing device measurements. The linear velocity of the moving velocity is the derivative of displacement over time or the integral of acceleration over time. By measuring the displacement of a flying metal body, the velocity signal can be obtained by differentiating it [16]. Measuring the speed of a moving object in a weapons test includes the flight speed of a typical flying metal body such as a projectile and the moving speed of the mechanical components. The speed of a flying metal body is often measured by the time interval, and the speed of a mechanical component is often measured by the instantaneous speed. Commonly used methods include coil targets, sky screen targets, photoelectric targets, acoustic targets, radar speed measurements, and optical fiber interferometer speed measurements.

The working principle of the coil target is based on the electromagnetic induction theory, and the object to be measured must be a magnet [17]. The skylight target takes the sky background as the target surface and forms a light curtain with a small thickness through the cracks behind the self-focusing lens. The receiving part receives all the light comprising the sky curtain. When the incoming target crosses the canopy, it causes a change in the light signal collected by the sensor, and the changes in the optical signal are converted into changes in the electrical signal through the photosensitive element, thus obtaining the measured information. During the use of the sky screen target, the changes in the light and shade of the background sky and the surrounding scenery in the light screen will lead to inaccurate detection. The photoelectric target is composed of the light source generating the light curtain and the photoelectric installation device. The invisible light curtain is used as the target surface. The difference between it and the skylight target is that it has its own light source and is not affected by the background light, so it is more suitable for indoor or underwater targets. The acoustic target is a device that converts the shock wave signal generated by the supersonic flight of projectiles into electrical signals through a microphone. Radar velocity measurements use the Doppler effect to measure the speed of a flying metal body [18].

The principle of optical fiber interferometer speed measurement is as follows. When light shines on the flying body, the flying body will change the frequency of the incident light. Interference occurs when the light reflected at different times reaches the detector at the same time. The motion characteristics of the flying body can be obtained by analyzing the interference fringe [19].

To sum up, the advantages and disadvantages of the commonly used detection methods for measuring the motion parameters of typical flying metal bodies such as artillery shells and rockets are shown in Table 1 [20]. The photoelectric and eddy current detection structure designed in this study is cheaper than the common conventional target detection systems while meeting the requirements of detection distance and time.

The modern mode of warfare is a high-tech confrontation between two or more parties in the battle controlled by intelligence. Aiming at the different characteristics of the incoming target group in the process from launch to flight to striking (or before striking), in light of the goals of light weight, modularity, and intelligence, in this study, the technology for detecting flying metal bodies based on eddy currents and photoelectric composite sensing was proposed. Compared with the methods of detection using active emission light sources such as optical fiber interferometers, lasers, and millimeter-wave radar commonly used in the modern battlefield, this technique has better concealment ability and weaker infrared characteristics when detecting incoming flying metal objects. The study also considered that the detection distance of the eddy current sensors used in the market is in the order of millimeters, and that the photoelectric conversion devices (their working principle is similar to that of skylight targets) will be affected by the background environment (changes in the light and shade in the background sky), which leads to the problem of unreliable detection. Therefore, further research was carried out on how to improve the detection range of the eddy current sensors, and infrared sensors without their own light source were adopted to detect an incoming target during high-speed flight.

### 3.4. Design of the Detection Circuit

The detection circuit included the photoelectric detection circuit and the eddy current detection circuit. The photoelectric detection circuit was built through the selection of appropriate sensors and designing the corresponding test circuit to carry out signal acquisition. The eddy current detection circuit was built by combining the coil parameters obtained from the simulation analysis, winding the coil to be used as the eddy current probe coil, designing the conditioning circuit, and completing the process of collecting signals.

**Figure 3:**
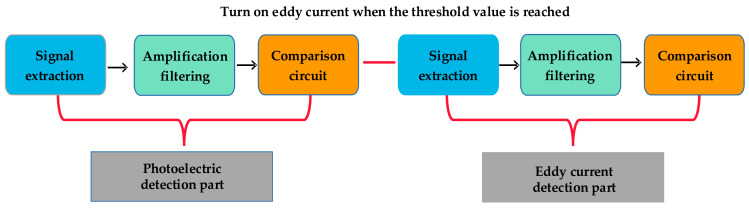
Circuit flow chart.

The photoelectric detection part uses the signal extraction circuit, the amplification and filtering circuit, and the comparison circuit to convert the infrared signals to electrical signals. The eddy current detection part also converts the moving target’s signal to electric signals through the signal extraction circuit, the amplification and filtering circuit, and the comparison circuit. A flow diagram of the circuit is shown in Figure 3.

#### 3.4.1. Design of the Photoelectric Detection Circuit

(1) Design of the signal extraction circuit

**Figure 4:**
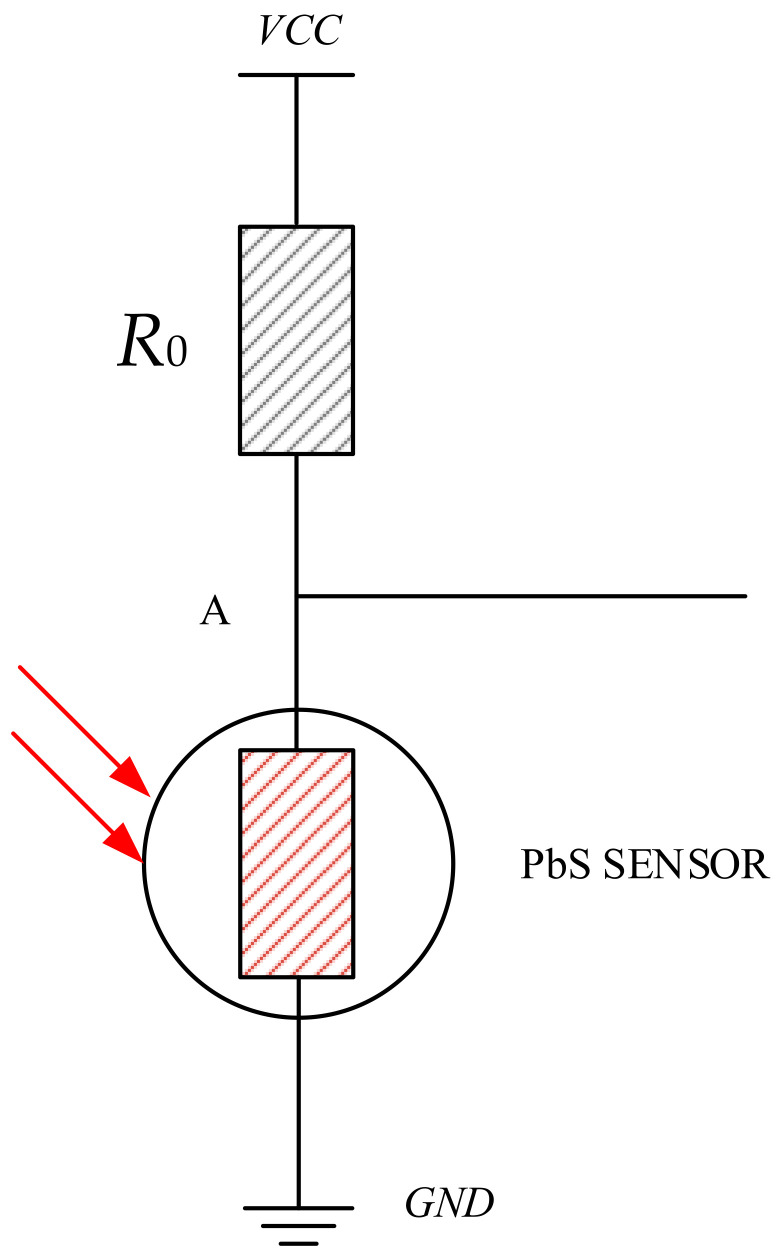
Photoelectric signal extraction circuit.

In order to detect the infrared signal of a flying metal body at a long distance, it is necessary to design the signal extraction circuit. In line with the working principle of a PbS sensor, the incident photons arriving at the polycrystalline film promotes electrons to pass through the band gap, thus improving the conductivity of the material and reducing the resistance of the material. The design of the photoelectric signal extraction circuit is shown in Figure 4.

In Figure 4, the detection range at a load resistance *R*_0_ and PbS detection are shown. The voltage is affected by the partial voltage at *R*_0_. The value of *R*_0_ was analyzed and calculated. Assuming that the resistance of the PbS sensor under illumination is *R_g_* and the dark resistance is *R_a_*, the range of the variation in voltage Δu at Point A is calculated by Formula (5). The sensor is in series. Under the action of VCC, when the resistance of the PbS sensor changes, the voltage drop at both ends of the load resistance can be measured to realize the conversion of the infrared signals to electrical signals. The range of variation in the voltage at Point A reflects the photoelectric effect.

(5) $$\Delta u=VCC\left(\frac{R_{a}}{R_{a}+R_{0}}-\frac{R_{g}}{R_{g}+R_{0}}\right)$$

where Δu is the range of the variation in voltage at Point A, *R*_0_ is the detection range at a load resistance, *R_g_* is the bright resistance of the PbS sensor, *R_a_* is the dark resistance.

Δu reflects the range of photoelectric detection, so the larger the Δu, the larger the detection range. The maximum value of Δu needs to be calculated. We obtain the maximum by taking the derivative of delta *u*.

(6) $$\frac{d\Delta u}{dR_{0}}=VCC\left(\frac{\left(R_{a}R_{g}-R_{0}{}^{2}\right)\times \left(R_{g}+R_{a}\right)}{{\left(R_{g}+R_{0}\right)}^{2}\times {\left(R_{a}+R_{0}\right)}^{2}}\right)$$

where Δu is the range of the variation in voltage at Point A, *R*_0_ is the detection range at a load resistance, *R_g_* is the bright resistance of the PbS sensor, *R_a_* is the dark resistance.

When the partial derivative of *R*_0_ with respect to Δu is taken, that is, when $R_{0}=\sqrt{R_{a}R_{g}^{}}$, Δu is maximized. The ideal *R*_0_ value can be obtained by the bright resistance *R_g_* and the dark resistance *R_a_* of the PbS sensor. In practical applications, *R*_0_ = *R_a_* = *R_g_* can be taken when infrared radiation does not make the sensor’s internal resistance change significantly. However, since the dark resistance of the PbS sensor is affected by temperature, the resistance value of *R*_0_ can be adjusted according to the context of application.

(2) Design of the signal amplification and filtering circuit

**Figure 5:**
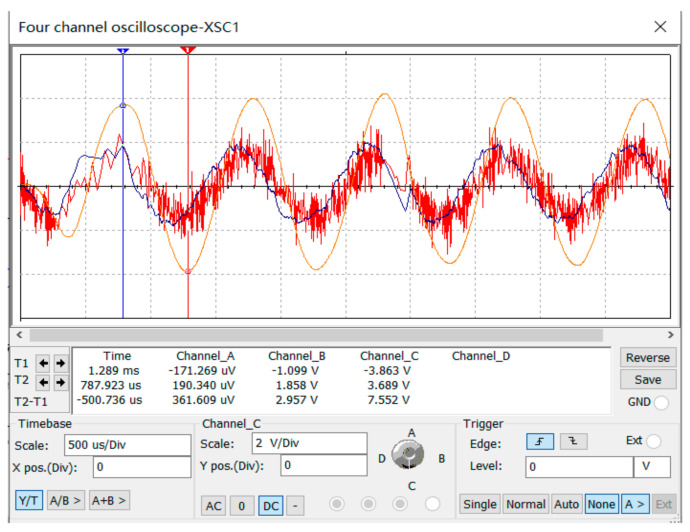
Detection circuit amplification and filtering simulation.

In order to use the infrared detector (PbS sensor) to detect a flying metal body at a relatively long distance, the signal collected by the sensor will be relatively weak when the distance is relatively long. If it is not amplified, the signal emitted by the flying body cannot be effectively detected. Therefore, it is necessary to amplify the signal collected by the sensor. In the design of the amplifier circuit, the DC component of the signal is first filtered by the straight divider capacitor. The in-phase amplifier circuit structure is used to improve the input resistance of the amplifier. In order to ensure that the amplification is not distorted, two stages are used to amplify the amplifier. A simulation of the amplification and filtering detection circuit is shown in Figure 5.

#### 3.4.2. Design of the Eddy Current Detection Circuit

**Figure 6:**
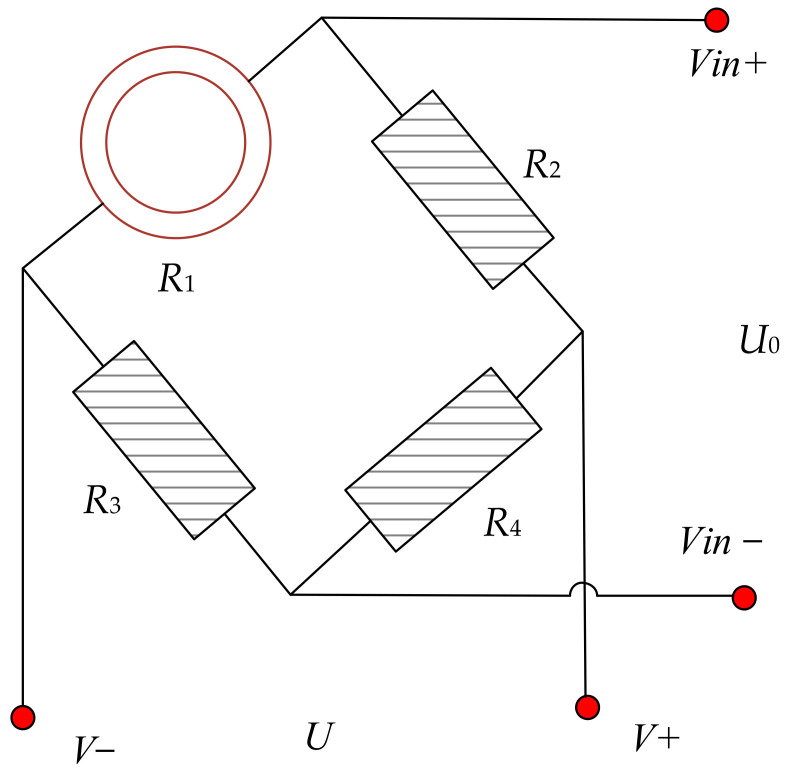
Eddy current signal extraction circuit.

The eddy current sensor converts the change in the nonelectrical parameters into changes in the electrical parameters through the conversion circuit for output. In this study, the DC bridge method was used to optimize the eddy current signal extraction circuit. The bridge method converts a weak change in the resistance value into a change in the electrical signal, which can effectively improve the accuracy of eddy current detection. The eddy current signal extraction circuit is shown in Figure 6.

In Figure 6, the winding coil is regarded as one arm of the bridge, and the other three arms use fixed resistance to convert the nonelectrical change of the eddy current coil into the electrical change (the output of the bridge). DC excitation is used as the bridge input. When the uniform magnetic field generated by the eddy current coil is slightly disturbed, the generated electromotive force changes the potential difference between the two ends of the coil (the output signal), which is multiplied by the bridge circuit.

**Figure 7:**
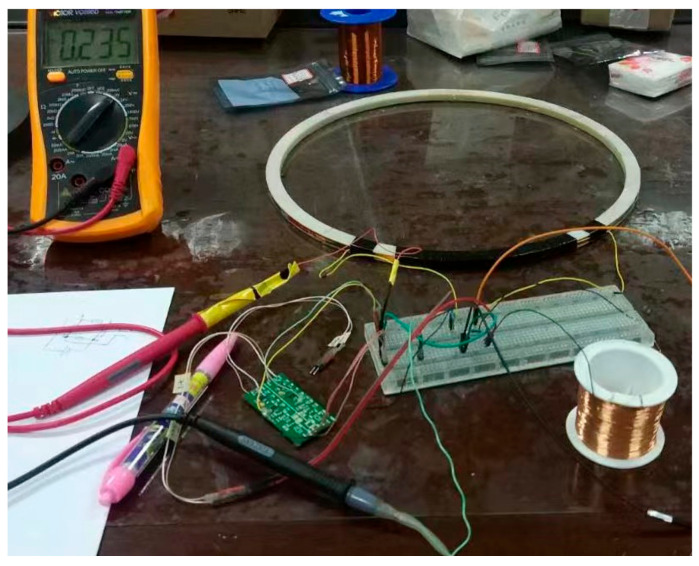
Eddy current coil structure.

In the bridge, the eddy current coil *R*_1_ can be regarded as having variable resistance, and its variation is denoted $\Delta R_{1}$. *R*_2_, *R*_3_ and *R*_4_ are fixed resistors, and *U*_0_ is the output voltage. The structure of the eddy current coil is shown in Figure 7. In the initial state, the bridge is in equilibrium. When the eddy current coil has a change in $\Delta R_{1}$, the bridge output voltage *U*_0_ is:

(7) $$U_{0}=\frac{\frac{R_{4}}{R_{3}}\times \frac{\Delta R_{1}}{R_{1}}}{\left(1+\frac{R_{3}}{R_{1}}+\frac{\Delta R_{1}}{R_{1}}\right)\times \left(1+\frac{R_{4}}{R_{3}}\right)}\times U$$

where *R*_1_ is the resistance of the eddy current coil, *R*_2_, *R*_3_, and *R*_4_ are fixed resistors, *U* is the input voltage, *U*_0_ is the output voltage.

#### 3.4.3. Calculation of the Response Time

The response time of the detection device is calculated by taking a typical metal projectile as the attack target (which enters the detection range vertically). The projectile’s flight speed is generally 600–1200 m/s. The maximum detection distance of the selected lead sulfide (PbS) sensor is 20 m, and its response time is less than 200 μs. The response time represents the speed of infrared detector from receiving the infrared radiation to making a response. According to the velocity time formula $t=\frac{s}{v}$, the projectile can fly for 16 ms within the detection range of the lead sulfide sensor. The response time of the lead sulfide infrared sensor is 200 μs, the response time of the conditioning circuit is 0.35 ms, and the running time of a single chip is 0.07114 ms. The total response time of the whole photoelectric detection circuit is about 620 μs. The response time of the photoelectric detection circuit is far less than the flight time of the projectile within the detection range of the lead sulfide sensor, so the designed photoelectric detection circuit can detect the flying metal body and carry out the response.

The response time of the eddy current sensor was less than 10 μs, the response time of the conditioning circuit was 0.35 ms, and the detection distance of the eddy current coil was 50 cm. According to the velocity time formula $t=\frac{s}{v}$, it was found that the projectile flew for 0.42 ms within the detection range of eddy current sensor, and the total response time of the whole eddy current detection component was about 0.36 ms. The response time of the eddy current detection circuit was less than the flight time of the projectile within the detection range of the eddy current sensor, so the designed eddy current detection system could detect the flying metal body and carry out the response.

In the eddy current simulation experiment, a metal plate was used as the target for detection. The response time of the eddy current sensor was less than 10 μs, the response time of the conditioning circuit was 0.35 ms, and the detection distance of the eddy current coil was 50 cm. According to the formula of velocity and time $t=\frac{s}{v}$, it was found that the movement time of the metal plate in the detection range of the eddy current sensor was 0.523 s, and the response time of the whole eddy current detection circuit was 0.36 ms. The response time of the eddy current detection circuit is much less than the time it takes for the metal plate to fly through the sensor, so the eddy current detection part in the simulation experiment could detect the metal plate and carry out the response.

**Figure 8:**
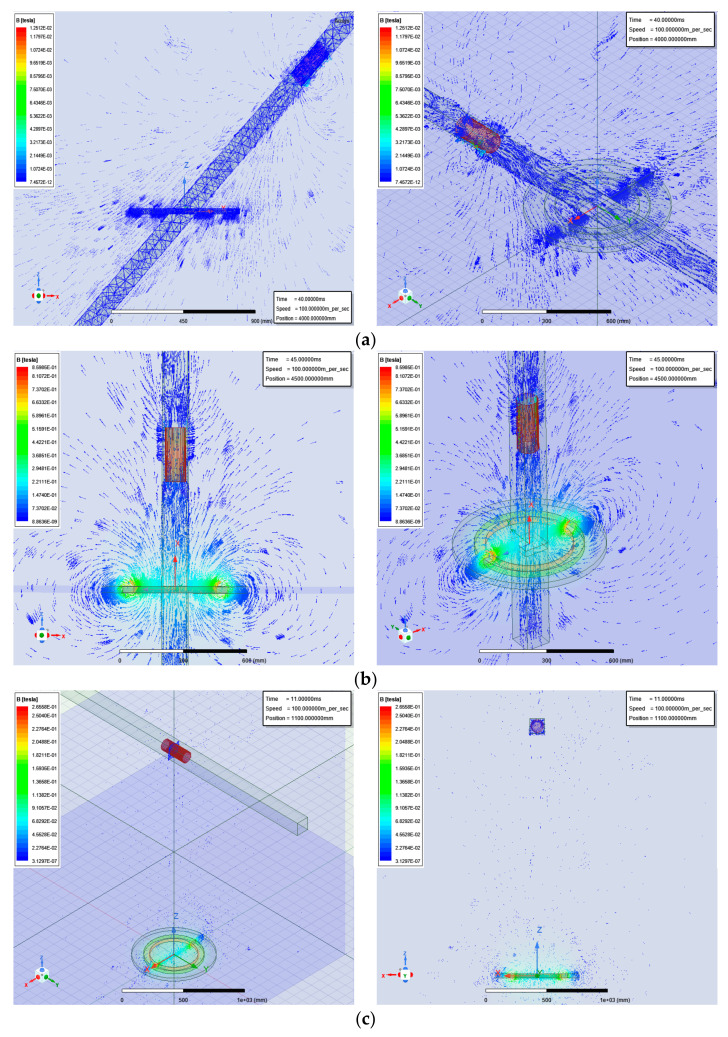
Simulation of eddy currents at different incidence angles. (**a**) 45°; (**b**) 90°; (**c**) 0°.

The effectiveness of the metal detection system was verified by simulating the flight trajectory of a flying metal body, setting different flight angles, and simulating the eddy current’s magnetic field in Maxwell. Simulated eddy currents at different incidence angles are shown in Figure 8.

## 4. Analysis of the Experiments

**Figure 9:**
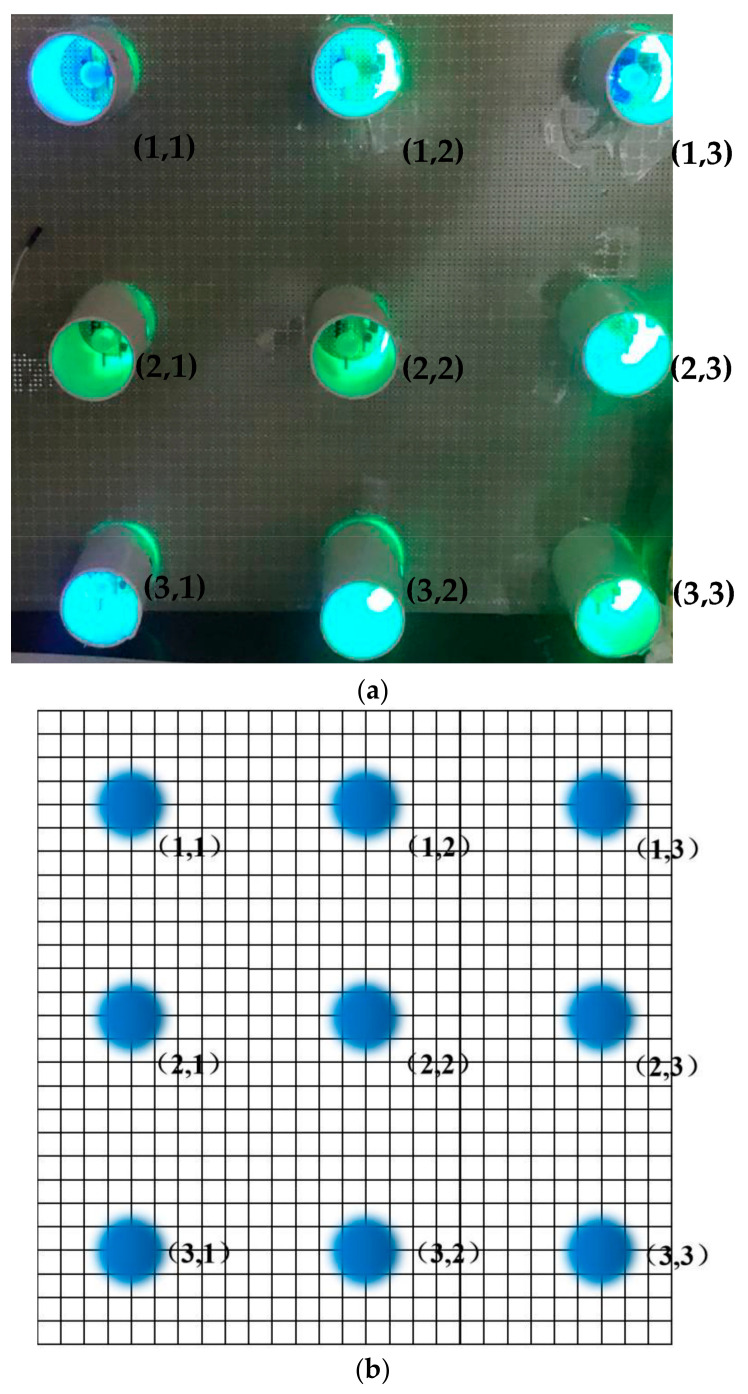
Photoelectric detection device. (**a**) Photoelectric detection experimental device; (**b**) equivalent diagram of photoelectric detection experimental device.

The physical nature of infrared radiation is that of thermal radiation. The degree of thermal radiation is mainly determined by the temperature of an object. The higher the temperature, the more infrared radiation is emitted, and the stronger the energy of the infrared radiation. The infrared radiation band is divided into four bands according to the wavelength: 0.76–3 μm for near infrared, 3–6 μm for middle infrared, 6–15 μm for far infrared, and 15–1000 μm for extreme far infrared. During the high-speed flight of a rocket and shell, both of the detection targets show the characteristics of medium-wave infrared radiation, so the use of a medium infrared detector is the best. However, in the actual simulation experiment, it is difficult to find the infrared object with mid-infrared waveform of radiation. In the simulated detection experiment, an infrared light source was used as the radiator. It was determined that the far infrared wavelength emitted by the radiator was around 9.6 μm. A photoelectric sensor as used as the detection component. The design of the photoelectric detection device is shown in Figure 9.

The response time of the electric sensor was about 300 ms, so a photodiode sensor was added to the pixel point. The back-end program used “or” logic, that is, if the photoelectric sensor or photodiode sensor had an output of 1, then the pixel output (judgment) was 1. The detection device was built 110 cm above the ground, and the infrared target was 170 cm, which ensured that the detection device could detect the infrared signal sent by the target.

At the beginning of the simulation experiment, the detection distance of the two sensors was measured. After measurement, the longest detection distance of the photoelectric sensor was 15 m, and the longest detection distance of the photodiode sensor was 5 m under ideal conditions. Precisely because a photosensitive diode cannot adapt to environmental light during detection, a simulation experiment was carried out on the combined photoelectric sensor and photosensitive diode. The infrared target moved in the direction (1,1) → (1,3) outside the detection range, and the detection results are shown in Table 2.

**Figure 10:**
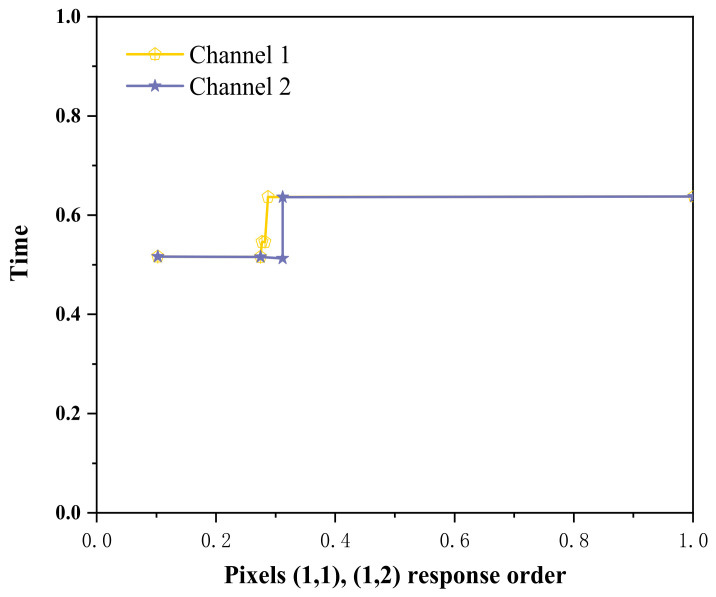
Response sequence of pixels (1,1) and (1,2).

Table 2, Table 3 and Table 4 represent each pixel from Points (1,1) to (3,3) in Figure 5, and ① to ③ represent the sequence of the pixels lighting up on the LCD screen. The light and dark order of Pixels ①–③ were captured by an oscilloscope. The response sequence of the pixels at Points (1,1) and (1,2) during detection of the infrared target is shown in Figure 10.

**Figure 11:**
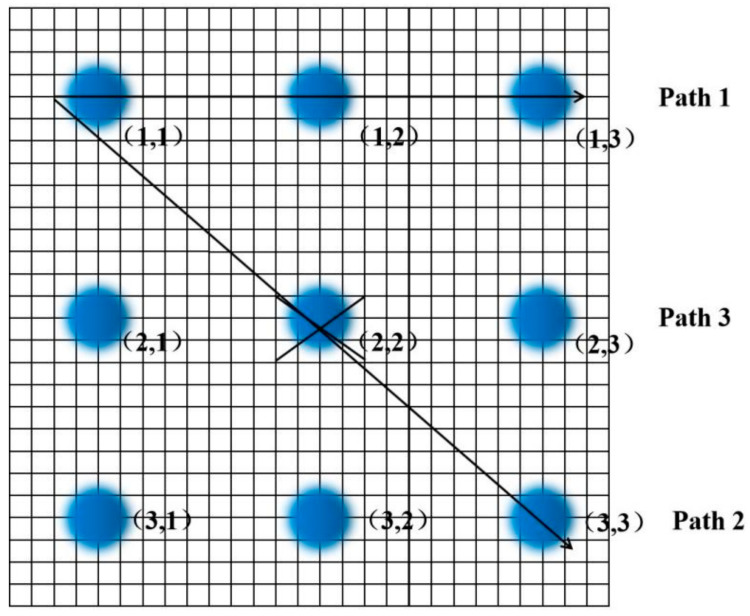
Target movement path equivalence diagram.

In Figure 10, Channel 1 (the red curve) is the action curve of the pixel at Point (1,1). Channel 2 (the blue curve) is the action curve of the pixel at Point (1,2). As can be seen from Figure 6, the time interval between the actions of the two pixels was about 40 ms, that is, the occlusion passed through the detection range of the photoelectric detection device at a certain speed, and the motion velocity of the occlusion could be obtained. The target movement path equivalence diagram is shown in Figure 11.

A small occlusion with a cross-sectional area of 10 cm moved along the preset path. According to the preset Path 1, it moved 5 m away from the photoelectric device, and the pixels were lit in the sequence (1,1) → (1,2) → (1,3). When it moved 5 m away from the photoelectric detection device according to preset Path 2, the pixels were lit in the sequence (1,1) → (2,2) → (3,3). According to Path 3, it gradually approached the photoelectric detection device from 5 m away, and the pixel at Points (2,2) was always on.

**Figure 12:**
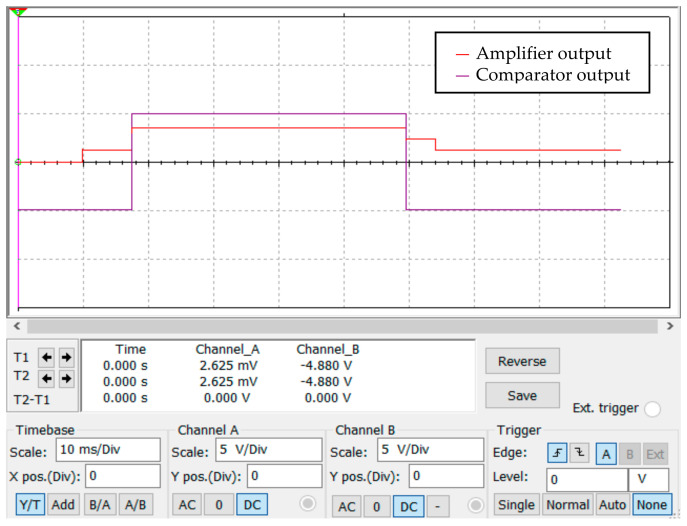
Optimized circuit simulation output diagram.

The output diagram of the optimized circuit simulation is shown in Figure 12. It can be seen from Figure 12 that the output of the comparator was low at the initial moment, the bridge was in balance, and the output signal was approximately 0 after passing through the amplifier. With a change in the resistance value of the impedance, the signal output of the amplifier became larger. When the response threshold had been reached, the comparator flipped and output a high-level signal (the response signal output of the eddy current sensor).

## 5. Conclusions

This study presented a method of detecting flying metal objects based on photoelectric and eddy current sensors. The problems in the process of detecting flying metal objects were analyzed, a detection framework based on photoelectric and eddy current sensors was designed according to the flight characteristics of flying metal targets, and a model of the photoelectric and eddy current sensors used for detection model was built. On this basis, the composite detection circuit structure and eddy current coil structure were optimized. The photoelectric and eddy current detection structure designed in this study is cheaper than the common conventional target detection systems while meeting the requirements of detection distance and time. The designed model was simulated and tested through electromagnetic field simulations and circuit simulations. The experimental results show that the framework and method of detecting flying metal objects based on composite photoelectric and eddy current sensing could meet the requirements of good detection and have practical value.

## Figures and Tables

**Table 1:** Comparison of detection methods.

Detection Method	Active and Passive	Detection Range	Volume	Price	Response Time
Inductive type	Ac magnetic field	0.001–1 mm	Small	Middle	<10 μs
Eddy current type	Variable magnetic field or fixed magnetic field	0–25 mm	Small	Middle	<10 μs
Photoelectric position sensing device	Laser as light source	35–690 mm	Big	Expensive	0.3–13 μs
Sky screen target	Against a background of natural light	4 m	Big	Expensive	<1 μs
Photoelectric target	Belt light source	12 m	Big	Expensive	<0.5 μs
Radar	Active emission electromagnetic wave	Kilometer level	Big	Expensive	<100 ms
Fiber optic interferometer	Active emission laser	10 m	Small	Expensive	0.16 μs
Design of this paper (Photoelectricity and eddy current)	Passive detection	Meter level	Small	Middle	<1 ms

**Table 2:** Moves along (1,1) → (1,3).

(1,1)	①	(1,2)	②	(1,3)	③
(2,1)	①	(2,2)	②	(2,3)	③
(3,1)	①	(3,2)	②	(3,3)	③

The infrared target moves in the direction (1,3) → (1,1) outside the detection range, and the detection results are shown in Table 3.

**Table 3:** Moves along (1,3) → (1,1).

(1,1)	③	(1,2)	②	(1,3)	①
(2,1)	③	(2,2)	②	(2,3)	①
(3,1)	③	(3,2)	②	(3,3)	①

The infrared target enters the detection range vertically from the detection distance, and the detection results are shown in Table 4.

**Table 4:** Vertical entry detection range.

(1,1)	①	(1,2)	①	(1,3)	①
(2,1)	①	(2,2)	①	(2,3)	①
(3,1)	①	(3,2)	①	(3,3)	①

## Data Availability

Not applicable.

## References

[1] Luo B., Wang H., Zhang Y., Chen J. (2022). Research on metal detector probe based on finite element simulation. Transducer Microsyst. Technol..

[2] Wang C., Guo W., Gao W., Ma T., Jin S. (2022). Research on metal target detection performance of large radius eddy current. J. Ordnance Equip. Eng..

[3] Li Y., Zhang Q., Liu X., Xiao N., Ning P., Li Y. (2022). Metal foreign body detection based on double/multiple differential coils pair magnetic module. J. Magn. Magn. Mater..

[4] Zheng Z., Jiang X., Luo Z. (2013). Design and analysis of metal detector based on eddy current effect. Exp. Sci. Technol..

[5] Lu M., Meng X., Huang R., Peyton A., Yin W. (2021). Analysis of tilt effect on notch depth profiling using thin-skin regime of driver-pickup eddy-current sensor. Sensors.

[6] Vyroubal D. (2009). Eddy-current displacement transducer with extended linear range and automatic tuning. IEEE Trans. Instrum. Meas..

[7] Liang B. (2018). Research and implementation of finite element simulation of eddy current sensor. Telecommun. Power Technol..

[8] He P., Wang Y., Fan L., Zuo W. (2020). Operation range analysis of the infrared detector for hypersonic flight vehicles. Laser Infrared.

[9] Lu L., Li H. (2020). A calculation model of infrared detection system with improved detection capability. Microw. Opt. Technol. Lett..

[10] Bailoo J.D., Bohlen M.O., Wahlsten D. (2010). The precision of video and photocell tracking systems and the elimination of tracking errors with infrared backlighting. J. Neurosci. Methods.

[11] Jiang X. (2011). Photoelectric Sensing and Detection Technology.

[12] Li H., Lei Z. (2013). Study and analysis on a new optical detection design method for photoelectric detection target. Sens. Rev..

[13] Man Y., Yang Q., Chen T. (2022). Infrared Single-Frame Small Target Detection Based on Block-Matching. Sensors.

[14] Lv Y. (2013). Research on Simulation Test of Infrared Radiation Characteristics of Flying Projectile. Master’s Thesis.

[15] Han X. (2020). Research on High Performance Displacement Sensor Based on Eddy Current Principle. Master’s Thesis.

[16] Yang L. (2020). Research on Sensing Technology of Eddy Current Displacement. Master’s Thesis.

[17] Wang K. (2015). Research on Velocity Measurement Technology of Coil Target. Master’s Thesis.

[18] Shang F., Kong D. (2018). Fundamentals of Weapon Testing Technology.

[19] Meng K. (2004). Optical Fiber Interferometry Technology.

[20] Chen D., Ni J., Bai L., Chen D. (2020). Detection method for the dynamic signal of sky screen-based velocity measurement system using Bayesian Generalized Likelihood Ratio Tests. Optik.

